# BEAM or cyclophosphamide in autologous haematopoietic stem cell transplantation for relapsing-remitting multiple sclerosis

**DOI:** 10.1038/s41409-024-02397-x

**Published:** 2024-08-26

**Authors:** Thomas Silfverberg, Christina Zjukovskaja, Yassine Noui, Kristina Carlson, AutoMS-Swe Investigators, Adjmal Nahimi, Ahlstrand Erik, Cherif Honar, Dreimane Arta, Einarsdottir Sigrun, Fagius Jan, Hägglund Hans, Iacobaeus Ellen, Lange Niclas, Lenhoff Stig, Ljungman Per, Lycke Jan, Mellergård Johan, Piehl Fredrik, Svenningsson Anders, Tolf Andreas, Joachim Burman

**Affiliations:** 1 Department of Medical Sciences, Uppsala University, Uppsala, Sweden; 2 Center for Clinical Research Dalarna, Uppsala University, Falun, Sweden; 3 Department of Clinical Sciences, Clinical Memory Research Unit, Faculty of Medicine, Lund University, Lund, Sweden; 4 Department of Medicine, Faculty of Medicine and Health, Örebro University, Örebro, Sweden; 5 Department of Hematology, Linköping University Hospital, Linköping, Sweden; 6 Institute of Medicine, University of Gothenburg, Gothenburg, Sweden; 7 Department of Hematology and Coagulation, Sahlgrenska University Hospital, Gothenburg, Sweden; 8 Department of Cellular Therapy and Allogeneic Stem Cell Transplantation, Karolinska University Hospital Huddinge, Karolinska Comprehensive Cancer Center, Stockholm, Sweden; 9 Department of Clinical Neuroscience, Karolinska Institutet, Stockholm, Sweden; 10 Department of Neurology, Karolinska University Hospital, Stockholm, Sweden; 11 Department of Hematology, Oncology & Radiophysics, Skåne University Hospital, Lund, Sweden; 12 Department of Medicine Huddinge, Karolinska Institutet, Stockholm, Sweden; 13 Department of Clinical Neuroscience, Institute of Neuroscience and Physiology, Sahlgrenska Academy, Gothenburg, Sweden; 14 Department of Neurology in Linköping, Linköping, Sweden; 15 Department of Biomedical and Clinical Sciences, Linköping University, Linköping, Sweden; 16 Department of Clinical Sciences and Department of Neurology, Danderyd Hospital, Karolinska Institutet Danderyd Hospital, Stockholm, Sweden

**Keywords:** Epidemiology, Stem-cell therapies, Chemotherapy

## Abstract

The most widely used conditioning regimens in autologous haematopoietic stem cell transplantation (ASCT) for multiple sclerosis (MS) are BEAM with anti-thymocyte globulin (ATG) and high-dose cyclophosphamide with ATG (Cy/ATG). In this retrospective study, we compare efficacy and safety of these regimens when used for relapsing-remitting MS. We assessed 231 patients treated in Sweden before January 1, 2020. The final cohort comprised 33 patients treated with BEAM/ATG and 141 with Cy/ATG. Prospectively collected data from the Swedish MS registry were used for efficacy, and electronic health records for procedure-related safety. The Kaplan–Meier estimate of ‘no evidence of disease activity’ (NEDA) at 5 years was 81% (CI 68–96%) with BEAM/ATG and 71% (CI 63–80%) with Cy/ATG, *p* = 0.29. Severe adverse events were more common with BEAM/ATG, mean 3.1 vs 1.4 per patient, *p* = <0.001. Febrile neutropaenia occurred in 88% of BEAM/ATG patients and 68% of Cy/ATG patients, *p* = 0.023. Average hospitalisation was 3.0 days longer in BEAM/ATG patients from day of stem-cell infusion, *p* < 0.001. While both regimens showed similar efficacy, BEAM/ATG was associated with more severe adverse events and prolonged hospitalisation. In the absence of randomised controlled trials, Cy/ATG may be preferable for ASCT in patients with relapsing-remitting MS due to its favourable safety profile.

## Introduction

Multiple sclerosis (MS) is considered an autoimmune disease of the central nervous system. Advances in therapeutic interventions targeting the immune system have improved the outcome for patients with MS. High-dose chemotherapy followed by autologous haematopoietic stem cell transplantation (ASCT) was developed to treat acute myeloid leukaemia in the 1970s and has become a cornerstone in the treatment of several haematological diseases. Encouraging results from animal experiments [1, 2], as well as case reports of patients with haematological disease and concurrent autoimmune disease treated with ASCT or allogeneic haematopoietic stem cell transplantation [3, 4], paved the way for its use in MS by the late 1990s [5, 6]. The efficacy and safety of ASCT for MS have improved in recent years, primarily due to better understanding of patient selection, conditioning regimens and increased centre experience [7]. As is the case for all disease-modifying treatments (DMTs), the evidence base is stronger for relapsing-remitting MS (RRMS) compared to progressive MS and, consequently, ASCT is mainly used for RRMS today [8–11].

Early studies of ASCT for MS utilised high-intensity conditioning regimens, which were associated with high toxicity and treatment-related mortality [12]. With time, the use of intermediate-intensity conditioning regimens have become dominant and safety has improved significantly [13]. In the last decade, two conditioning regimens have primarily been used: the myeloablative BEAM and the immunoablative (non-myeloablative) cyclophosphamide protocol (Cy). Both are typically given alongside T-cell depleting serotherapy, most commonly anti-thymocyte globulin (ATG). The use of BEAM/ATG has remained stable whereas the use of Cy/ATG has increased in recent years [8]. Both conditioning regimens induce high rates of clinical remission in patients with RRMS, but it remains unclear how these compare against each other [14, 15].

Using data from the observational study Haematopoietic Stem Cell Transplantation for Treatment of Multiple Sclerosis in Sweden (AutoMS-Swe) [16], we compared efficacy and safety of BEAM/ATG and Cy/ATG for RRMS.

## Materials/subjects and methods

### Data collection

The Swedish MS registry (SMSreg) and local European Society for Blood and Marrow Transplantation (EBMT) registries were used to identify patients. SMSreg has extensive, nationwide coverage of prospectively collected data on diagnosis, progress and treatment of MS going back almost 30 years [17]. A neurologist scrutinised the data with electronic patient records in order to validate its accuracy, including disease course.

Haematologists retrospectively collected safety data from electronic patient records from the start of conditioning until 100 days after ASCT. The Common Terminology Criteria for Adverse Events (CTCAE) version 5.0 was used to grade all severe adverse events (AEs) [18]. Haematological cytopaenias, transient alopecia and amenorrhoea were expected during the first weeks after ASCT and were not included as AEs in the analysis. A comprehensive list of data points has been published previously [16].

### Inclusion criteria

A diagnosis of MS according to the revised McDonald criteria [19], with a relapsing-remitting disease course and ASCT performed for MS in Sweden before January 1, 2020, was required for inclusion.

### Exclusion criteria

The exclusion criteria consisted of a diagnosis of primary or secondary progressive MS or clinically isolated syndrome, according to Lublin et al, [20] at the time of ASCT or a failure to meet minimal data requirements. These requirements were defined as data on disease course at the time of transplantation, conditioning regimen, date of transplantation, and at least one follow-up visit (unless early death before first follow-up visit) with data on clinical assessment using the Kurtzke expanded disability status scale (EDSS) [21] and radiological assessment with magnetic resonance imaging (MRI).

### Endpoints

The primary endpoints were the Kaplan–Meier estimate of the composite *no evidence of disease activity* (NEDA) 5 years after ASCT and treatment-related mortality. Secondary endpoints were Kaplan–Meier estimates of NEDA at 3 and 10 years, progression-free survival, relapse-free survival, MRI event-free survival and confirmed disability worsening (CDW) at 3, 5 and 10 years. Additional secondary endpoints were the annualised relapse rate (ARR) after ASCT, the EDSS change between baseline and follow-up at 1, 2 and 3 years, and the proportion of patients with clinical improvement. Procedure-related safety endpoints were the frequency and grade of severe AEs. A more thorough description of the definitions of variables and outcomes has been published previously [16].

### Procedures

#### Mobilisation

Cyclophosphamide 2 g/m^2^ and granulocyte colony-stimulating factor (G-CSF) 5 micrograms/kg subcutaneously daily from day 5 or 6 until stem cell harvest were used to mobilise the stem cells.

#### Harvest

Stem cell harvest was performed by apheresis of peripheral blood to a minimal yield of 2.0 × 10^6^ CD34+ cells/kg. The stem cells were cryopreserved with no ex-vivo manipulation.

#### Conditioning

The 7-day BEAM/ATG protocol included carmustine (BCNU) 300 mg/m^2^ on day −7, etoposide 100 mg/m^2^ twice daily on day −6 to −3 (in total 800 mg/m^2^), cytarabine arabinoside 800 mg/m^2^ continuous infusion day −6 to −3, melphalan 140 mg/m^2^ on day −2, and ATG from rabbit (thymoglobulin) 5 mg/kg on day +1 to +2 (in total 10 mg/kg). Minimum washout time was 48 h until reinfusion of the harvested stem cells.

The 5-day Cy/ATG protocol included cyclophosphamide 50 mg/kg on day −5 to −2 (in total 200 mg/kg) and thymoglobulin, 0.5 mg/kg day −5, 1 mg/kg day −4 and 1.5 mg/kg day −3 to −1 (in total 6 mg/kg). Additionally, 1000 mg IV methylprednisolone was administered day −5 to −1 (total 5000 mg) including tapering for 7 days from 30 mg/day on day 0. Hyperhydration and uromitexan (MESNA) were administered day −5 to −2 to prevent haemorrhagic cystitis. Minimum washout time was 24 h.

#### Antimicrobial prophylaxis

During the neutropaenic phase, the patients received oral ciprofloxacin to prevent bacterial infection, except for eight patients who received prophylactic intravenous antibiotics. Prophylaxis against herpes viruses and *Pneumocystis jiroveci* was prescribed for a minimum of three months following ASCT. Reactivation of cytomegalovirus (CMV) and Epstein-Barr virus (EBV) was monitored for patients with positive serology according to local routines.

#### Supportive care

Any administered blood products were filtered and irradiated until the lymphocyte counts exceeded 1.0 × 10^9^/L.

### Statistical analysis

Statistical analysis was performed with R V.4.2.3 (using the packages: ggplot2, survival, fBasics, ggpubr, moments, survimner, plotrix, grid, gridExtra, cowplot, tidyverse and devtools). Data were summarised using frequencies for categorical variables, medians and interquartile range (IQR) for discrete variables and means and standard deviations (±SD) for continuous variables. Frequencies were presented with a 95% confidence interval (95% CI). To determine statistically significant differences between two time points, the Wilcoxon signed rank test was used. Differences in time to progression, relapse, confirmed disability worsening, new MRI event or death were estimated using the log-rank test in Kaplan–Meier plots. Unpaired Student’s t-tests were used to determine statistical significance for means and proportions of normal distribution. A two-tailed *p* < 0.05 was considered statistically significant.

## Results

### Patient inclusion

We identified 231 patients with MS that were treated with ASCT. Fifteen patients did not meet the inclusion criteria. Another 42 patients fulfilled at least one exclusion criterion. These 57 patients were not analysed further, and the final cohort consisted of 33 patients treated with BEAM/ATG and 141 patients treated with Cy/ATG. Data were exported from SMSreg on May 22, 2022, corresponding to the last date of follow-up.

### Patient characteristics

**Fig. 1:**
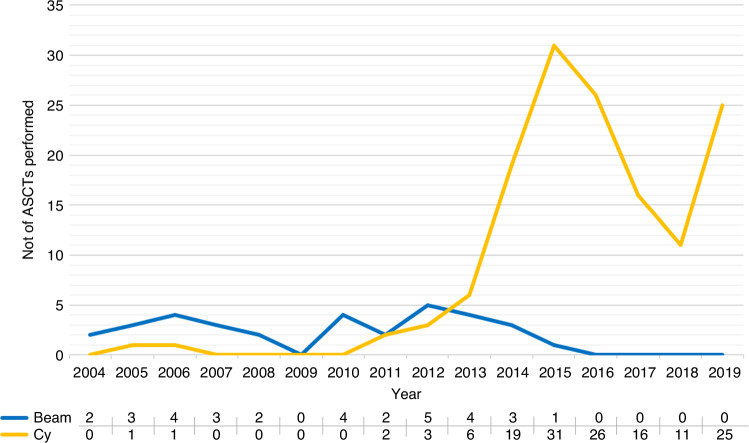
The number of patients treated with BEAM/ATG or Cy/ATG per year in Sweden. Number of autologous hematopoietic stem cell transplantations (ASCTs) performed per year in Sweden for relapsing-remitting multiple sclerosis separated by conditioning regimen.

The first patient was treated on May 25, 2004, and the last patient on November 26, 2019. The average follow-up time was longer with BEAM/ATG than Cy/ATG, mean 10 (±2.5) vs 5.0 (±2.4) years, *p* < 0.0001; see Fig. 1. Baseline patient characteristics (Table 1) were balanced in terms of age, sex, number of previous DMTs and comorbidities. Disability at the time of ASCT was higher in patients treated with BEAM/ATG compared to Cy/ATG (median 4.0 vs 3.0, *p* = 0.002). The proportion of patients with gadolinium-enhancing lesions on MRI at baseline was higher in patients treated with BEAM/ATG than Cy/ATG (66.6% vs 44.0%, *p* = 0.02) as well as the ARR (median 3.1 vs 1.3, *p* = 0.002). See Table 1 for details.

**Table 1:** Patient characteristics.

*n* = 174	BEAM/ATG (%) *n* = 33	Cy/ATG (%) *n* = 141	*P* value
Age (years, range 9.0–58.8)			0.94^e^
0–9	-	1 (0.7)	
10–19	2 (6.1)	4 (2.8)	
20–29	14 (42.4)	56 (39.0)	
30–39	12 (36.4)	56 (39.0)	
40–49	5 (15.2)	21 (14.9)	
50–59	-	3 (2.1)	
Sex			
Female/Male	19 (57.6)/14 (42.4)	93 (66.0)/48 (34.0)	0.22^f^
Comorbidities^a^			
Depression	1 (3.0)	6 (4.3)	
Obesity	1 (3.0)	4 (2.8)	
Asthma	-	5 (3.5)	
Bipolar disorder	2 (6.1)	2 (1.4)	
Anxiety disorder	-	4 (2.8)	
Crohn’s disease	1 (3.0)	2 (1.4)	
Hypertension	1 (3.0)	2 (1.4)	
Psoriasis	-	3 (2.1)	
Prior malignancy^b^	1 (3.0)	1 (0.7)	
Diabetes mellitus	1 (3.0)	1 (0.7)	
Chronic renal disease	1 (3.0)	1 (0.7)	
Rheumatoid arthritis	1 (3.0)	1 (0.7)	
Prior deep vein thrombosis	-	2 (1.4)	
Thyrotoxicosis	-	2 (1.4)	
Ankylosing spondylitis	2 (6.1)	-	
Irritable bowel syndrome	-	2 (1.4)	
No comorbidity	24 (72.7)	98 (69.5)	
Prior disease modifying treatment			
None	4 (12.1)	14 (9.9)	
Interferon	5 (15.2)	8 (5.7)	
Natalizumab	12 (36.4)	42 (29.8)	
Mitoxantrone	5 (15.2)	0	
Glatiramer acetate	4 (12.1)	5 (3.5)	
Intravenous immunoglobulin	1 (3.0)	1 (0.7)	
Fingolimod	0	13 (9.2)	
Dimethyl fumarate	0	66 (46.8)	
Teriflunomide	0	4 (2.8)	
Rituximab	2 (6.1)	44 (31.2)	
Alemtuzumab	0	4 (2.8)	
Prior lines of treatment	2 IQR (1–3)	2 IQR (1–2)	0.72^e^
Naïve	4 (12.1)	14 (9.9)	
1	7 (21.2)	43 (30.5)	
2	14 (42.4)	42 (29.8)	
3	8 (24.2)	19 (13.5)	
4	0	14 (9.9)	
5	0	8 (5.7)	
6	0	1 (0.7)	
EDSS at ASCT^c^	4 IQR (3–6)	3 IQR (2–4)	0.002^e^
0–1.5	2 (6.1)	21 (14.9)	
2–3.5	11 (33.3)	77 (54.6)	
4–5.5	11 (33.3)	27 (19.1)	
6–6.5	6 (18.2)	10 (7.1)	
7–9.5	3 (9.1)	5 (3.5)	
Gadolinium-enhancing lesions at ASCT ^d^	22 (66.7)	62 (44.0)	0.02^**f**^
0	11 (33.3)	79 (56.0)	
1–9	5 (15.2)	40 (28.4)	
10–20	3 (9.1)	8 (5.7)	
>20	8 (24.2)	5 (3.5)	
Years since MS diagnosis	3.8 ± 3.0	4.8 ± 4.7	0.67^e^
ARR in the year prior to ASCT	3.1 ± 3.2	1.3 ± 1.3	0.002^e^

Demographics and characteristics of the study cohorts.

*EDSS* Kurtzke expanded disability status scale, *ARR* annualised relapse rate.

^a^Comorbidities with a frequency of more than 1%,

^b^Two cases of breast cancer.

^c^Data missing for one patient.

^d^Fifteen patients did not have a contrast-enhanced MRI scan at baseline.

^e^Mann–Whitney’s test.

^f^Fisher’s test.

### In-patient care

The median interval between administration of cyclophosphamide at mobilisation and start of conditioning was 26.3 (±15.3) days for BEAM/ATG and 36.8 (±19.8) for Cy/ATG. The average duration of hospitalisation after stem cell infusion was longer in patients treated with BEAM/ATG than Cy/ATG (16.8 ± 5.8 vs 13.8 ± 2.7 days), *p* < 0.001.

The average time to engraftment (defined as absolute neutrophil count (ANC) > 0.5 × 10^9^/L and platelets >20 × 10^9^/L and rising without transfusion) was 11.6 days (±3.0) in the BEAM/ATG group compared to 12.4 (±2.3) in the Cy/ATG group, *p* = 0.095. The average time to ANC > 0.5 × 10^9^/L was shorter in the BEAM/ATG group than in the Cy/ATG group (11.0 days ±3.1 vs 12.3 days ±2.1), *p* = 0.005. G-CSF was used during the neutropaenic phase in 3 (9.1%) patients treated with BEAM/ATG and in 26 (18%) patients treated with Cy/ATG, *p* = 0.19.

The average weight loss was 2.5 kilograms (±2.4) in patients treated with BEAM/ATG and 2.1 (±2.0) in patients treated with Cy/ATG, *p* = 0.33. The average decrease in plasma albumin was greater in patients treated with BEAM/ATG than Cy/ATG, 10 (±5.3) vs 7.4 (±4.1) g/L, *p* < 0.002.

### Primary endpoints

**Fig. 2:**
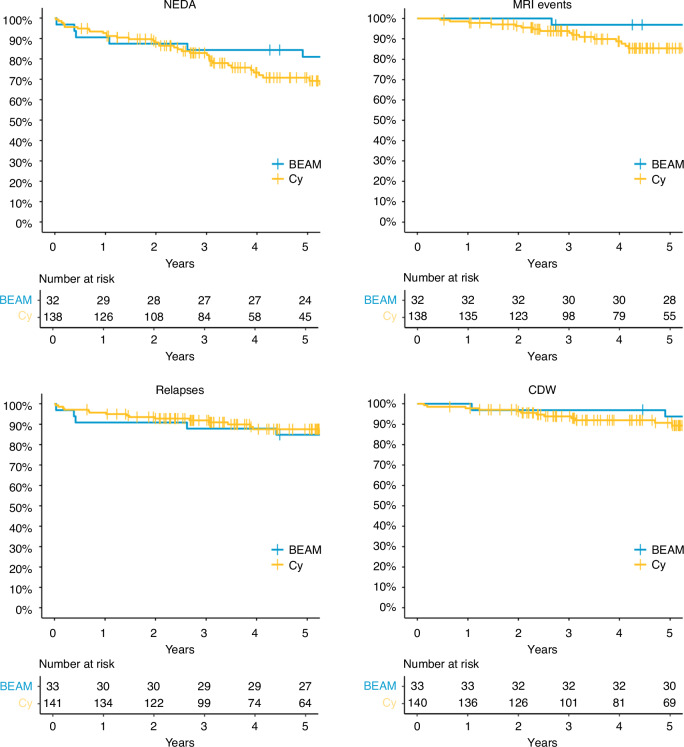
Kaplan–Meier estimates of NEDA, and freedom from relapse, new MRI events and CDW. Kaplan–Meier estimates of maintaining the composite endpoint no evidence of disease activity (NEDA), including the Kaplan–Meier estimates of the individual parts of NEDA: freedom from new MRI events, freedom from relapse and freedom from confirmed disease worsening (CDW) after autologous haematopoietic stem cell transplantation (ASCT) up to 5 years of follow-up.

There was no statistically significant difference in the Kaplan–Meier estimate of NEDA at 5 years, which was 81% (95%, CI 68–96%) in the BEAM/ATG cohort and 71% (95%, CI 63–80%) in the Cy/ATG cohort, *p* = 0.29, see Fig. 2 and Table 2. There was no treatment-related mortality in either of the cohorts.

**Table 2:** Secondary efficacy endpoints.

*n* = 174	BEAM/ATG *n* = 33			Cy/ATG*n* = 141		
	*n* at risk	KM	95% CI	*n* at risk	KM	95% CI
NEDA						*p* = 0.11^b^
1 year	29	91%	81%–100%	126	93%	89%–97%
2 years	28	88%	77%–100%	108	88%	83%–94%
3 years	27	84%	73%–98%	84	83%	77%–90%
5 years	24	81%	68%–96%	45	71%	63%–80%
10 years	12	78%	64%–94%	1	58%	45%–74%
Freedom from new MRI event^a^						p = 0.08^b^
1 year	32	100%	100%	135	99%	97%–100%
2 years	32	100%	100%	123	96%	93%–100%
3 years	30	97%	91%–100%	98	94%	90%–98%
5 years	28	97%	91%–100%	55	85%	79%–92%
10 years	14	88%	76%–100%	2	77%	65%–90%
Freedom from clinical relapses						p = 0.99^b^
1 year	30	91%	82%–100%	134	96%	92%–99%
2 years	30	91%	82%–100%	122	93%	89%–97%
3 years	29	88%	77%–100%	99	92%	87%–97%
5 years	27	85%	73%–98%	64	88%	82%–94%
10 years	13	85%	73%–98%	3	86%	79%–93%
Freedom from CDW						p = 0.44^b^
1 year	33	97%	91%–100%	136	98%	95%–100%
2 years	32	97%	91%–100%	126	96%	94%–100%
3 years	32	97%	91%–100%	101	94%	90%–98%
5 years	30	94%	85%–100%	69	91%	86%–96%
10 years	16	90%	81%–100%	2	87%	81%–95%

Kaplan–Meier probability for NEDA and its parameters following ASCT.

*KM* Kaplan–Meier estimate of probability, *NEDA* No Evidence of Disease Activity, *CDW* Confirmed Disease Worsening, *MRI* Magnetic Resonance Imaging

^a^Data are missing for 1 BEAM/ATG and 3 Cy/ATG patients.

^b^Log-rank test.

### Secondary endpoints

#### Kaplan–Meier estimates

The secondary efficacy endpoints of NEDA (Kaplan–Meier estimates at 3 and 10 years) and absence of clinical relapses, new MRI lesions and CDW are visualised in Fig. 2 and listed in Table 2. There was no significant difference in the proportion of patients maintaining NEDA, with clinical relapses, MRI events or CDW. The ARR after ASCT was comparable between the two groups: BEAM/ATG 0.015 ± 0.044 and Cy/ATG 0.033 ± 0.11, *p* = 0.97.

#### Disability

**Fig. 3:**
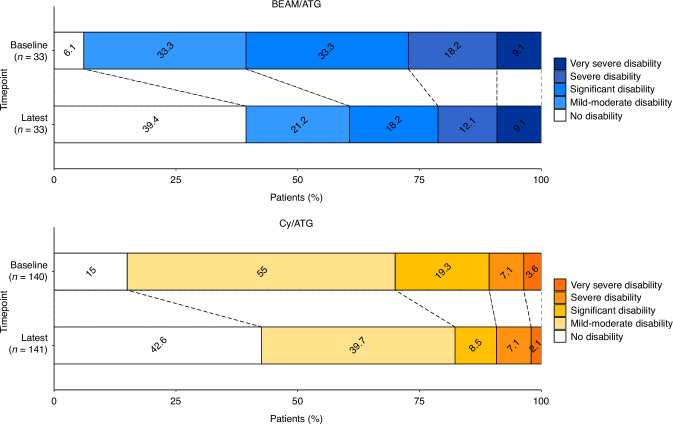
Proportions of patients with disability at baseline and last follow-up. Burden of disability measured as proportions of patients with different levels of disability at baseline and last follow-up after autologous haematopoietic stem cell transplantation (ASCT) with BEAM/ATG and Cy/ATG.

CDW, based on the last available EDSS value compared to EDSS at baseline, occurred in 3 (9.1% CI −0.72–19%) patients treated with BEAM/ATG and 14 (9.9% CI 5.0–15%) patients treated with Cy/ATG, *p* = 0.88. The corresponding number of patients with confirmed disability improvement was 18 (55% CI 38–72%) in the BEAM/ATG group and 69 (49% CI 40–57%) in the Cy/ATG group, *p* = 0.56. The median overall change in EDSS was a decrease by 1 point in both groups (IQR 0–2), see Fig. 3.

#### Long-term follow-up

There were 3 (9.1% CI −0.72–19%) BEAM/ATG-treated patients who received additional DMT after ASCT compared to 17 (12% CI 6.7–17%) Cy/ATG-treated patients, p = 0.63. One (3.0%) patient treated with BEAM/ATG received rituximab compared to 12 (8.5%) patients treated with Cy/ATG, *p* = 0.28.

Conversion to secondary progressive MS occurred in 6 (18%) patients in the BEAM/ATG group after an average of 4.9 (±3.2) years and in 6 (4.3%) patients in the Cy/ATG group after an average of 3.5 (±1.8) years, *p* = 0.37.

### Safety

#### General

The average number of all severe AEs per patient were 3.1 (±1.8) in the BEAM/ATG group and 1.4 (±1.2) in the Cy/ATG group, *p* < 0.001. The average numbers of grade 4 AEs were 0.15 (±0.36) and 0.043 (±0.29) respectively, *p* = 0.065. Two patients (6.1%) in the BEAM/ATG cohort did not experience any severe AEs compared to 28 patients (20%) treated with Cy, *p* = 0.059. Febrile neutropaenia occurred more often in the BEAM/ATG group compared to the Cy/ATG group; 29 (88%, CI 77–99%) vs 96 (68%, CI 60–76%), *p* = 0.023. Severe AEs that were more common in the BEAM/ATG group and were highly statistically significant included serum sickness, hypokalaemia, hypoalbuminaemia, diarrhoea and anorexia (defined as significant weight loss or malnutrition resulting in enteral tube feeding or total parenteral nutrition). Pericarditis, atrial fibrillation, elevated transaminases and hyperglycaemia occurred only in patients treated with Cy/ATG. All severe AEs are listed in Table 3.

**Table 3:** Severe adverse events.

	BEAM/ATG (%) *n* = 33		Cy/ATG (%) *n* = 141		P value	Total (%) *n* = 174	
	Grade 3	Grade 4	Grade 3	Grade 4		Grade 3	Grade 4
Febrile neutropaenia^a^	26 (78.8)	3 (9.1)	93 (66.0)	3 (2.1)	0.023	119 (68.4)	6 (3.4)
Hypokalaemia^b^	14 (43.8)	-	17 (12.1)	-	<0.0001	31 (17.8)	-
Nausea	5 (15.2)	-	9 (6.4)	-	0.095	14 (8.0)	-
Serum sickness	7 (21.2)	-	4 (2.8)	-	<0.0001	11 (6.3)	-
Oral mucositis	4 (12.1)	-	5 (3.5)	-	0.044	9 (5.1)	-
Diarrhoea	5 (15.2)	-	4 (2.8)	-	0.0037	9 (5.1)	-
Elevated AST/ALT	-	-	9 (6.4)	-	0.14	9 (5.1)	-
Hypoalbuminaemia	7 (21.2)	-	-	-	<0.0001	7 (4.0)	-
Hypotension	2 (6.1)	-	4 (2.8)	-	0.35	6 (3.4)	-
Fatigue	1 (3.0)	-	4 (2.8)	-	0.95	5 (2.9)	-
Anorexia	4 (12.1)	-	1 (0.7)	-	0.0024	5 (2.9)	-
Hyperglycaemia	-	-	5 (3.5)	-	0.28	5 (2.9)	-
Thromboembolic event	1 (3.0)	-	1 (0.7)	1 (0.7)		2 (1.1)	1 (0.6)
Pericarditis	-	-	2 (1.4)	1 (0.7)		2 (1.1)	1 (0.6)
Depression	-	1 (3.0)	2 (1.4)	-		2 (1.1)	1 (0.6)
Cytokine release syndrome	2 (6.1)	-	1 (0.7)	-		3 (1.7)	-
Pneumonia (non-neutropaenic)^c^	2 (6.1)	-	1 (0.7)	-		3 (1.7)	-
Skin/soft tissue infection (non-neutropaenic)^c^	1 (3.0)	-	2 (1.4)	-		3 (1.7)	-
Catheter-related infection (non-neutropaenic)^c^	-	-	3 (2.1)	-		3 (1.7)	-
Vascular access thrombosis	1 (3.0)	-	2 (1.4)	-		3 (1.7)	-
Myalgia	1 (3.0)	-	2 (1.4)	-		3 (1.7)	-
Vomiting	1 (3.0)	-	2 (1.4)	-		3 (1.7)	-
Elevated gamma-GT^d^	1 (4.7)	-	2 (1.7)	-		3 (2.1)	-
Heart failure	-	-	1 (0.7)	1 (0.7)		1 (0.6)	1 (0.6)
Hyponatremia	1 (3.0)	1 (3.0)	-	-		1 (0.6)	1 (0.6)
Sepsis (non-neutropaenic)^c^	2 (6.1)	-	-	-		2 (1.1)	-
Infectious enterocolitis (non-neutropaenic)^c^	1 (3.0)	-	1 (0.7)	-		2 (1.1)	-
Seizures	1 (3.0)	-	1 (0.7)	-		2 (1.1)	-
Fever (non-neutropaenic)^c^	2 (6.1)	-	-	-		2 (1.1)	-
Urticaria	1 (3.0)	-	1 (0.7)	-		2 (1.1)	-
Syncope	1 (3.0)	-	1 (0.7)	-		2 (1.1)	-
Abdominal pain	1 (3.0)	-	1 (0.7)	-		2 (1.1)	-
Postherpetic pain	1 (3.0)	-	1 (0.7)	-		2 (1.1)	-
CMV reactivation	-	-	1 (0.7)	-		1 (0.6)	-
EBV reactivation	-	-	1 (0.7)	-		1 (0.6)	-
Varicella zoster	-	-	1 (0.7)	-		1 (0.6)	-
Pyelonephritis (non-neutropaenic)^c^	-	-	1 (0.7)	-		1 (0.6)	-
Non-infectious enterocolitis	1 (3.0)	-	-	-		1 (0.6)	-
Atrial fibrillation	-	-	1 (0.7)	-		1 (0.6)	-
Hypoxia	-	-	1 (0.7)	-		1 (0.6)	-
Pulmonary infiltrates	-	-	1 (0.7)	-		1 (0.6)	-
Interstitial oedema in lungs	-	-	1 (0.7)	-		1 (0.6)	-
Acute kidney injury	-	-	1 (0.7)	-		1 (0.6)	-
Allergic reaction	-	-	1 (0.7)	-		1 (0.6)	-
Leukocytosis	-	-	1 (0.7)	-		1 (0.6)	-
Immunologic thrombocytopenia	-	-	1 (0.7)	-		1 (0.6)	-
Mania	-	-	1 (0.7)	-		1 (0.6)	-
Hallucinations	1 (3.0)	-	-	-		1 (0.6)	-
Hemichorea	-	-	1 (0.7)	-		1 (0.6)	-
Vaginal haemorrhage	-	-	1 (0.7)	-		1 (0.6)	-
Elevated ALP^e^	-	-	1 (0.8)	-		1 (0.6)	-

All grade 3 and 4 adverse events according to CTCAE v5.0 for all patients from start of mobilisation to day +100 after ASCT. Anaemia, neutropaenia, leukopaenia and thrombocytopaenia as well as transient alopecia and amenorrhoea were expected during the first weeks after ASCT and were excluded. Neurological adverse events assessed as manifestations of MS were not included. There were no grade 5 adverse events. *P* values shown for variables with five or more events.

^a^Febrile neutropaenia comprises all episodes of fever (according to CTCAE v5.0) regardless of clinical infection occurring during the neutropenic phase following stem cell mobilisation and conditioning.

^b^Hypokalaemia was associated with furosemide treatment after hyperhydration in 15 patients; 2 BEAM/ATG and 13 Cy/ATG patients. Data were missing for 1 BEAM/ATG patient.

^c^Occurring outside the neutropaenic phase.

^d^Data were missing for 12 BEAM/ATG and 20 Cy/ATG patients.

^e^Data were missing for 5 BEAM/ATG and 12 Cy/ATG patients.

#### Overall mortality

One patient in the BEAM/ATG group died during the follow-up period. The death occurred in a patient with pre-existing depression and previous suicide attempts and was due to suicide more than six years after ASCT. The death was deemed unrelated to the ASCT.

#### Intensive care

There was no statistically significant difference in the proportions of patients who received intensive care, *p* = 0.23. Two patients in the BEAM/ATG group (6.1%) were admitted to an intensive care unit; due to hyponatraemia, one of them had repetitive seizures. Three (2.1%) patients in the Cy/ATG cohort needed intensive care, due to sepsis and hypoxia (*n* = 1), sepsis with hypotension (*n* = 1), and pulmonary embolism in combination with perimyocarditis with transient heart failure (left ventricle ejection fraction 30%) and pericardial effusion (*n* = 1).

#### Bacterial infections

Bacterial infections verified by culture or unequivocal clinical picture were more common in the BEAM/ATG group, affecting 21 patients (64%, CI 47–80%) compared to 40 (28%, CI 21–36%) patients in the Cy/ATG group, *p* = 0.0001. All 33 patients in the BEAM/ATG group were treated with intravenous broad-spectrum antibiotics, on average 11 days (±4.8), compared to 106 patients (75%) in the Cy/ATG group, on average 9.3 days (±3.7), *p* = 0.0013. Eight patients in the Cy/ATG group received prophylactic intravenous antibiotics instead of oral ciprofloxacin. Sepsis or septicaemia were more common in patients treated with BEAM/ATG, 14 (42%, CI 26–59%) vs 7 (5.0%, CI 1.4–8.5), *p* < 0.0001. The most commonly found bacterial species were *Escherichia coli* and streptococci in both cohorts.

Notably, there were no cases of EBV- or CMV-related disease, invasive fungal infection, haemorrhagic cystitis or death due to COVID-19 in either cohort. A description of the reported infections is presented in Table 4.

**Table 4:** Infections.

*n* = 174	BEAM/ATG (%) *n* = 33	Cy/ATG (%) *n* = 141	*P* value
Verified bacterial species			
*E. coli*	7 (21)	14 (9.9)	0.077
*Enterococci sp*.	6 (18)	-	<0.0001
*Streptococci sp*.	7 (21)	6 (4.3)	
Coagulase-negative *staphylococci*	3 (9.1)	4 (2.8)	
*Staphylococci sp*.	-	2 (1.4)	
*K. pneumoniae*	3 (9.1)	1 (0.71)	
*C. difficile*	2 (6.1)	2 (1.4)	
*P. aeriginosa*	2 (6.1)	-	
Clinical fungal infections			
Oral candidiasis	1 (3.0)	3 (2.1)	
Vaginosis	1 (3.0)	-	
Verified viral infections			
Herpes zoster	2 (6.1)	1 (0.71)	
Herpes simplex	2 (6.1)	1 (0.71)	
BK-polyoma^a^	-	4 (2.8)	
Influenza	-	2 (1.4)	
Other respiratory viruses	1 (3.0)	5 (3.5)	
Calici	-	1 (0.71)	
Parvovirus B19	-	1 (0.71)	
CMV^b^			
Detectable viral DNA	8 (27)	41 (29)	
Persistently measurable in at least two samples	2 (6.7)	6 (4.3)	
Oral treatment		5 (3.6)	0.29
IV treatment	-	1 (0.72)	
EBV^b^			
Detectable viral DNA	8 (27)	51 (37)	0.23
Persistently measurable in at least two samples	1 (3.3)	20 (14)	0.096
IV treatment	-	1 (0.72)	

All verified infections from start of conditioning until day +100 after ASCT for BEAM/ATG and Cy/ATG conditioning.

^a^One case detected in blood, all four in urine, but there were no cases of haemorrhagic cystitis.

^b^Data are missing for five patients.

## Discussion

In this retrospective study, we compared the two most widely used conditioning regimens in ASCT for treatment of RRMS: BEAM/ATG and Cy/ATG. The analysis of prospectively collected efficacy data from SMSreg revealed no statistically significant difference in the primary endpoint: the Kaplan–Meier estimate of NEDA at 5 years. However, the BEAM/ATG regimen was associated with a statistically significant higher incidence of severe AEs, including febrile neutropaenia, septicaemia/sepsis and bacterial infections.

Both conditioning regimens were associated with a high proportion of patients maintaining NEDA following ASCT, and there was no statistically significant difference between the groups at any timepoint. In addition, there was no statistically significant difference in the proportion of patients who were free from new MRI events. Taken together, these results suggest that there is no major difference in the efficacy of these two conditioning regimens. Similarly, the proportion of patients that did not have new relapses or worsened in disability were equal. There was a trend favouring BEAM/ATG in freedom from MRI events, however. It is possible that a study with larger sample sizes would be able to demonstrate a statistically significant difference in freedom from MRI events and the related NEDA outcome. It is questionable if a single instance of a new MRI lesion has clinical relevance and we conclude that our findings suggest that there is no major difference in efficacy between these two conditioning regimens.

Severe AEs were more commonly observed after BEAM/ATG, particularly infectious complications such as febrile neutropaenia, sepsis and septicaemia; the need for intravenous antibiotics; and verified bacterial infections. This is likely due to the higher intensity of the BEAM/ATG conditioning regimen. The rates of anorexia, diarrhoea and hypokalaemia were also higher with BEAM/ATG, which could be due to a more significant impact on mucous membranes. The greater toxicity of BEAM was also evident in the more considerable decrease in plasma albumin levels. BEAM was associated with higher gastrointestinal toxicity such as oral mucositis, diarrhoea and hypokalaemia, which likely explains the higher frequency of infections with *E. coli* and enterococci. In contrast, hyperglycaemia requiring intermittent insulin only occurred in patients treated with Cy/ATG, likely due to the high doses of methylprednisolone used in this protocol. A few cases of severe AEs typically associated with cyclophosphamide, such as pericarditis and elevated liver transaminases [22, 23], were observed and were not seen in patients with BEAM/ATG. Patients treated with BEAM/ATG were, on average, hospitalised 3 days longer from the day of stem cell infusion, probably due to toxicity in some patients keeping them hospitalised longer after engraftment.

Overall, the findings in this study correspond to what Hamerschlak and colleagues reported in a retrospective Brazilian study of 41 patients from 2010, where 90% of the patients had progressive MS. They observed comparable event-free survival between the cohorts, but noted more severe AEs, longer hospitalisation and three deaths in the BEAM/ATG cohort compared to Cy/ATG [24]. Additionally, in two reports from a retrospective EBMT study there was no difference between BEAM/ATG and Cy/ATG in time to engraftment, treatment-related mortality, NEDA, relapses or disability worsening in patients with RRMS [25, 26]. From a recent Danish retrospective study of 32 RRMS-patients, it was also reported higher toxicity with BEAM/ATG in comparison to Cy/ATG, but no evidence that BEAM/ATG is more effective than Cy/ATG [27].

There are contradictory reports on the risk of secondary malignancies after ASCT for MS. A Swedish retrospective study did not find any elevated risk [28], but in 2017 Muraro and colleagues reported a 3.2% risk of secondary malignancies including 1.1% risk of myelodysplastic syndrome [29]. Additional evidence suggests an elevated risk of myelodysplastic syndrome and acute myeloid leukaemia in individuals treated with alkylating agents in other settings. The risk has been reported to be more pronounced in melphalan-based regimens compared to those using cyclophosphamide [30, 31], with indications of a dose-response relationship [32]. While these increased risks have not been explicitly verified in MS patients, and constitutes findings from individual studies, it is prudent to exercise caution when selecting a conditioning regimen for MS, particularly considering the generally young demographics of these patients. On the other hand, high-dose cyclophosphamide has a dose-related association with cardiomyopathy. Clinically relevant toxicity is rare with the doses currently used [33], but include substantial and often refractory congestive heart failure with onset the first 10 days after ASCT [34]. In a case-control study of late debutant congestive heart failure following haematopoietic stem cell transplantation, high-dose cyclophosphamide was not associated with a higher risk of cardiotoxicity [35].

There were some noteworthy differences between the two groups, which may have influenced the outcome. A distinct shift in the preference for conditioning regimens over time was observed, potentially affecting outcomes in various ways. BEAM/ATG was the preferred regimen until 2011–2013, when Cy/ATG started to gain momentum. After 2015, BEAM/ATG was no longer used. It is possible that increased experience and improvements in standards of care could have led to less severe AEs and thereby an overestimation of the differences between BEAM/ATG and Cy/ATG. However, the pattern of more toxicity and longer hospitalisation was also apparent in the years 2011 to 2015 when the regimens were used in parallel. Over time, the use of ASCT also increased and the indication in RRMS broadened. Initially, it was considered a rescue treatment, reserved for patients with the most aggressive forms of MS, but the indication was later changed to include patients with active disease despite an adequate course of treatment. This is reflected in a lower proportion of patients with gadolinium-enhancing lesions and a lower ARR at baseline in the Cy/ATG-treated patients. It is not clear whether or how this would affect outcome, but it may have masked a superior efficacy of the more intensive regimen. The low number of patients, disparity in group sizes and low number of events are significant limitations and it is possible that a larger study would have detected more differences than the present one. The low number of events also made it impossible to conduct more sophisticated analyses, such as multivariable Cox regression.

In summary, we found no statistically significant difference between BEAM/ATG and Cy/ATG in the primary efficacy endpoint, the Kaplan–Meier estimate of NEDA at 5 years, or in the primary safety endpoint of treatment-related mortality. BEAM/ATG was associated with a higher frequency of severe adverse events and longer hospitalisation. It should be recognised that the two cohorts were not directly comparable since over time the indication for ASCT and also treatments for RRMS have changed. Thus, it is difficult to draw any firm conclusions regarding efficacy. In the absence of randomised controlled trials, our findings, along with previous reports, nevertheless suggest that Cy/ATG is the preferable option for ASCT in patients with relapsing-remitting MS due to its favourable safety profile.

## Data Availability

The study is registered at ClinicalTrials.gov with identification number NCT05029206. De-identified individual participant data supporting the findings presented in this article, including text, tables, figures, and will be accessible alongside the study protocol for a period of 5 years, starting at the time of the article’s publication. Researchers with a sound proposal without commercial purposes, can request access to this data by contacting joachim.burman@uu.se. To obtain access, those requesting data will need to sign a data access agreement.

## References

[1] Ikehara S, Good RA, Nakamura T, Sekita K, Inoue S, Oo MM, et al. Rationale for bone marrow transplantation in the treatment of autoimmune diseases. Proc Natl Acad Sci USA. 1985;82:2483–7. 3887403 10.1073/pnas.82.8.2483 PMC397583

[2] Ikehara S, Yasumizu R, Inaba M, Izui S, Hayakawa K, Sekita K, et al. Long-term observations of autoimmune-prone mice treated for autoimmune disease by allogeneic bone marrow transplantation. Proc Natl Acad Sci USA. 1989;86:3306–10. 2654943 10.1073/pnas.86.9.3306 PMC287120

[3] McAllister LD, Beatty PG, Rose J. Allogeneic bone marrow transplant for chronic myelogenous leukemia in a patient with multiple sclerosis. Bone Marrow Transpl. 1997;19:395–7. 10.1038/sj.bmt.1700666 9051253

[4] Yin JA, Jowitt SN. Resolution of immune-mediated diseases following allogeneic bone marrow transplantation for leukaemia. Bone Marrow Transpl. 1992;9:31–3. 1543947

[5] Burt RK, Traynor AE, Pope R, Schroeder J, Cohen B, Karlin KH, et al. Treatment of autoimmune disease by intense immunosuppressive conditioning and autologous hematopoietic stem cell transplantation. Blood. 1998;92:3505–14. 9808541

[6] Fassas A, Anagnostopoulos A, Kazis A, Kapinas K, Sakellari I, Kimiskidis V, et al. Peripheral blood stem cell transplantation in the treatment of progressive multiple sclerosis: first results of a pilot study. Bone Marrow Transpl. 1997;20:631–8. 10.1038/sj.bmt.1700944 9383225

[7] Das J, Sharrack B, Snowden JA. Autologous haematopoietic stem cell transplantation in multiple sclerosis: a review of current literature and future directions for transplant haematologists and oncologists. Curr Hematol Malig Rep. 2019;14:127–35. 30828772 10.1007/s11899-019-00505-z PMC6510794

[8] Sharrack B, Saccardi R, Alexander T, Badoglio M, Burman J, Farge D, et al. Autologous haematopoietic stem cell transplantation and other cellular therapy in multiple sclerosis and immune-mediated neurological diseases: updated guidelines and recommendations from the EBMT Autoimmune Diseases Working Party (ADWP) and the Joint Accreditation Committee of EBMT and ISCT (JACIE). Bone Marrow Transpl. 2020;55:283–306. 10.1038/s41409-019-0684-0 PMC6995781 31558790

[9] Burt RK, Han X, Quigley K, Helenowski IB, Balabanov R. Real-world application of autologous hematopoietic stem cell transplantation in 507 patients with multiple sclerosis. J Neurol. 2022;269:2513–26. 34633525 10.1007/s00415-021-10820-2 PMC8503710

[10] Casanova B, Jarque I, Gascón F, Hernández-Boluda JC, Pérez-Miralles F, de la Rubia J, et al. Autologous hematopoietic stem cell transplantation in relapsing-remitting multiple sclerosis: comparison with secondary progressive multiple sclerosis. Neurol Sci. 2017;38:1213–21. 28396953 10.1007/s10072-017-2933-6 PMC5489620

[11] Mancardi GL, Sormani MP, Di Gioia M, Vuolo L, Gualandi F, Amato MP, et al. Autologous haematopoietic stem cell transplantation with an intermediate intensity conditioning regimen in multiple sclerosis: the Italian multi-centre experience. Mult Scler. 2012;18:835–42. 22127896 10.1177/1352458511429320

[12] Sormani MP, Muraro PA, Schiavetti I, Signori A, Laroni A, Saccardi R, et al. Autologous hematopoietic stem cell transplantation in multiple sclerosis: a meta-analysis. Neurology. 2017;88:2115–22. 28455383 10.1212/WNL.0000000000003987

[13] Burt RK, Marmont A, Oyama Y, Slavin S, Arnold R, Hiepe F, et al. Randomized controlled trials of autologous hematopoietic stem cell transplantation for autoimmune diseases: the evolution from myeloablative to lymphoablative transplant regimens. Arthritis Rheum. 2006;54:3750–60. 17133541 10.1002/art.22256

[14] Burt RK, Balabanov R, Han X, Sharrack B, Morgan A, Quigley K, et al. Association of nonmyeloablative hematopoietic stem cell transplantation with neurological disability in patients with relapsing-remitting multiple sclerosis. JAMA. 2015;313:275–84. 25602998 10.1001/jama.2014.17986

[15] Nash RA, Hutton GJ, Racke MK, Popat U, Devine SM, Steinmiller KC, et al. High-dose immunosuppressive therapy and autologous HCT for relapsing-remitting MS. Neurology. 2017;88:842–52. 28148635 10.1212/WNL.0000000000003660 PMC5331868

[16] Silfverberg T, Zjukovskaja C, Ljungman P, Nahimi A, Ahlstrand E, Dreimane A, et al. Haematopoietic stem cell transplantation for treatment of relapsing-remitting multiple sclerosis in Sweden: an observational cohort study. J Neurol Neurosurg Psychiatry. 2024;95:125–33. 37748927 10.1136/jnnp-2023-331864 PMC10850659

[17] Hillert J, Stawiarz L. The Swedish MS registry—clinical support tool and scientific resource. Acta Neurol Scand. 2015;132:11–9. 26046553 10.1111/ane.12425 PMC4657484

[18] Common Terminology Criteria for Adverse Events (CTCAE) v5.0: US Department of Health and Human Services, National Institutes of Health, National Cancer Institute; 2017. Available from: https://ctep.cancer.gov/protocoldevelopment/electronic_applications/docs/ctcae_v5_quick_reference_5x7.pdf.

[19] Thompson AJ, Banwell BL, Barkhof F, Carroll WM, Coetzee T, Comi G, et al. Diagnosis of multiple sclerosis: 2017 revisions of the McDonald criteria. Lancet Neurol. 2018;17:162–73. 29275977 10.1016/S1474-4422(17)30470-2

[20] Lublin FD, Reingold SC, Cohen JA, Cutter GR, Sorensen PS, Thompson AJ, et al. Defining the clinical course of multiple sclerosis: The 2013 revisions. Neurology. 2014;83:278–86. 24871874 10.1212/WNL.0000000000000560 PMC4117366

[21] Kurtzke JF. Rating neurologic impairment in multiple sclerosis: an expanded disability status scale (EDSS). Neurology. 1983;33:1444–52. 6685237 10.1212/wnl.33.11.1444

[22] Emadi A, Jones RJ, Brodsky RA. Cyclophosphamide and cancer: golden anniversary. Nat Rev Clin Oncol. 2009;6:638–47. 19786984 10.1038/nrclinonc.2009.146

[23] McDonald GB, Slattery JT, Bouvier ME, Ren S, Batchelder AL, Kalhorn TF, et al. Cyclophosphamide metabolism, liver toxicity, and mortality following hematopoietic stem cell transplantation. Blood. 2003;101:2043–8. 12406916 10.1182/blood-2002-06-1860

[24] Hamerschlak N, Rodrigues M, Moraes DA, Oliveira MC, Stracieri AB, Pieroni F, et al. Brazilian experience with two conditioning regimens in patients with multiple sclerosis: BEAM/horse ATG and CY/rabbit ATG. Bone Marrow Transpl. 2010;45:239–48. 10.1038/bmt.2009.127 19584827

[25] Saccardi R, Badoglio M, Burman J, Helbig G, Kazmi MA, Mancardi G, et al. BEAM vs cyclophosphamide-based conditioning regimen in aggressive multiple sclerosis: a retrospective analysis of European Blood and Marrow Transplantation Society. Blood. 2019;134:3313.

[26] Saccardi R, Badoglio M, Burman J, Helbig G, Innocenti C, Kazmi MA, et al. BEAM vs cyclophosphamide-based conditioning regimen in aggressive multiple sclerosis: a retrospective analysis of European Blood and Marrow Transplantation Society. Bone Marrow Transpl. 2020;55:37–8.

[27] Jespersen F, Petersen SL, Andersen P, Sellebjerg F, Magyari M, Sørensen PS, et al. Autologous hematopoietic stem cell transplantation of patients with aggressive relapsing-remitting multiple sclerosis: Danish nation-wide experience. Mult Scler Relat Disord. 2023;76:104829. 37364374 10.1016/j.msard.2023.104829

[28] Alping P, Burman J, Lycke J, Frisell T, Piehl F. Safety of alemtuzumab and autologous hematopoietic stem cell transplantation compared to noninduction therapies for multiple sclerosis. Neurology. 2021;96:e1574–e84. 33514645 10.1212/WNL.0000000000011545 PMC8032381

[29] Muraro PA, Pasquini M, Atkins HL, Bowen JD, Farge D, Fassas A, et al. Long-term outcomes after autologous hematopoietic stem cell transplantation for multiple sclerosis. JAMA Neurol. 2017;74:459–69. 28241268 10.1001/jamaneurol.2016.5867 PMC5744858

[30] Greene MH, Harris EL, Gershenson DM, Malkasian GD Jr, Melton LJ 3rd, Dembo AJ, et al. Melphalan may be a more potent leukemogen than cyclophosphamide. Ann Intern Med. 1986;105:360–7. 3740675 10.7326/0003-4819-105-3-360

[31] Curtis RE, Boice JD Jr., Stovall M, Bernstein L, Greenberg RS, Flannery JT, et al. Risk of leukemia after chemotherapy and radiation treatment for breast cancer. NEJM. 1992;326:1745–51. 1594016 10.1056/NEJM199206253262605

[32] Godley LA, Larson RA. Therapy-related myeloid leukemia. Semin Oncol. 2008;35:418–29. 18692692 10.1053/j.seminoncol.2008.04.012 PMC2600445

[33] Murdych T, Weisdorf DJ. Serious cardiac complications during bone marrow transplantation at the University of Minnesota, 1977–1997. Bone Marrow Transpl. 2001;28:283–7. 10.1038/sj.bmt.1703133 11535997

[34] Goldberg MA, Antin JH, Guinan EC, Rappeport JM. Cyclophosphamide cardiotoxicity: an analysis of dosing as a risk factor. Blood. 1986;68:1114–8. 3533179

[35] Armenian SH, Sun C-L, Francisco L, Steinberger J, Kurian S, Wong FL, et al. Late congestive heart failure after hematopoietic cell transplantation. J Clin Oncol. 2008;26:5537–43. 18809605 10.1200/JCO.2008.17.7428 PMC2651101

